# Is Multidirectional UV Exposure Responsible for Increasing Melanoma Prevalence with Altitude? A Hypothesis Based on Calculations with a 3D-Human Exposure Model

**DOI:** 10.3390/ijerph13100961

**Published:** 2016-09-28

**Authors:** Michael Schrempf, Daniela Haluza, Stana Simic, Stefan Riechelmann, Kathrin Graw, Gunther Seckmeyer

**Affiliations:** 1 Institute of Meteorology and Climatology, Leibniz Universität Hannover, Hannover 30419, Germany; Seckmeyer@muk.uni-hannover.de; 2 Institute of Environmental Health, Center for Public Health, Medical University of Vienna, Vienna 1090, Austria; daniela.haluza@meduniwien.ac.at; 3 Institute of Meteorology, University of Natural Resources and Applied Life Sciences, Vienna 1190, Austria; stana.simic@boku.ac.at; 4 Physikalisch-Technische Bundesanstalt (PTB), Braunschweig 38116, Germany; stefan.riechelmann@ptb.de; 5 Deutscher Wetterdienst, Offenbach 63067, Germany; Kathrin.Graw@dwd.de

**Keywords:** UV radiation, human exposure, erythema, malignant melanoma, altitude effects, albedo, snow cover, alpine region

## Abstract

In a recent study, melanoma incidence rates for Austrian inhabitants living at higher altitudes were found to increase by as much as 30% per 100 m altitude. This strong increase cannot simply be explained by the known increase of erythemally-weighted irradiance with altitude, which ranges between 0.5% and 4% per 100 m. We assume that the discrepancy is partially explainable by upwelling UV radiation; e.g., reflected by snow-covered surfaces. Therefore, we present an approach where the human UV exposure is derived by integrating incident radiation over the 3D geometry of a human body, which enables us to take upwelling radiation into account. Calculating upwelling and downwelling radiance with a radiative transfer model for a snow-free valley and for snow-covered mountain terrain (with albedo of 0.6) yields an increase in UV exposure by 10% per 100 m altitude. The results imply that upwelling radiation plays a significant role in the increase of melanoma incidence with altitude.

## 1. Introduction

Cumulative life-time exposure to natural and artificial UV radiation is associated with chronic skin damage, including skin cancer. Many epidemiological studies prove the fundamental role of UV radiation in the genesis of skin cancer [1,2,3,4,5], of which the most hazardous form is malignant melanoma. Appearing in 1300 cases in 2009 in Austria, it accounted for 3.5% of all malignant tumors [6]. In studies of the increase of melanoma incidence (e.g., by Krishnamurthy [7] in India and by Gerbaud et al. [8] in France) the authors investigated local cancer registries and state that altitude may have an impact on melanoma incidence. Madera et al. [9] conducted a study in the province of Granada, Spain to assess the relationship between altitude, daily erythemal dose, and the prevalence of melanoma, and found a tendency toward increased prevalence of melanoma at higher altitude. A few recent studies focused on skin health and environmental factors in Austria. Haluza et al. [10] published a comprehensive study on Austrian melanoma incidence and mortality data, investigating its relation to the most important biological and environmental indices, such as gender, age, home district, and altitude. The authors found a significant increase of melanoma incidence with altitude in the Austrian districts with about 50% higher rates in urban compared to rural districts. Moehrle and Garbe [11] showed that the Swiss cancer registries and the Austrian Tyrol registry have much higher incidence rates for cutaneous melanoma than other Central European cancer registries. The authors hypothesize that mountaineering activities in higher altitude may increase the risk for cutaneous melanoma. 

As has been shown from numerous radiative transfer studies, UV irradiances change with the observing altitude due to changes in scattering and absorption [12]. The increase of UV irradiance with altitude cannot be simply described by one number, as it is a complex function of altitude, cloudiness, aerosol content, tropospheric ozone absorption, and snow cover [13]. Nevertheless, the percentage increase with altitude is often used as a proxy. Blumthaler et al. [14] measured an increase of the erythemal effective irradiance with altitude of 1.8% per 100 m. Similar values were given by other studies (e.g., Herman et al. [12], McKenzie et al. [15], Cordero et al. [16]), which led to the assumption that there is an upper limit of the increase with altitude, since no values above 4% per 100 m were reported.

Aside from existing studies for Austrian conditions (e.g., Schauberger et al. [17], Schmalwieser et al. [18,19]), a variety of exposure measurements on different subgroups can be found in the literature. Due to different methodological approaches, findings reported in these studies are not directly comparable. To address this issue, Seckmeyer et al. [20] presented a novel method to calculate vitamin-D-weighted exposure by integrating the incident solar spectral radiance over all relevant parts of the human body. It should be noted that individual UV exposure strongly depends on behavioral patterns, and is defined by the duration of the exposure, geometry of the receiving surface, by protection of clothing, the use of sunscreen, hair cover, and shadowing (e.g., Seckmeyer et al. [20], Weihs et al. [21], Haluza et al. [22]). Behavioral patterns are influenced by occupational activities, spare time activities, and the choice of holiday destinations [23]. In regard to recreational activities at higher altitudes, surface reflectance is important, especially in seasonally snow-covered and mountainous regions. The albedo of snow-covered surfaces may vary between 0.02 and 1 [24]. However, it is known that the albedo is influenced by a large surface area of more than 50 km from the observation point [25,26], where rocks, trees, streets and buildings with a lower reflectivity within that range may exist. Therefore, the effective albedo is usually much lower than unity [27,28,29]. Effective albedo values for the UV range have previously been determined through a combination of radiative transfer modeling and a 3D albedo model [27,29,30], averaging to 0.41 (aged snow) and 0.77 (fresh snow) for a 1000 m snowline. An effective albedo of 0.63 to 0.78 determined for the snowline at 800 m by Simic et al. [31] is comparable to the model calculations presented by Weihs et al. [29]. In Austria, Rengarajan et al. [30] measured the albedo at the Sonnblick observatory in winter. Their experimentally-determined values in the range of 0.73 to 0.78 are quite similar to the albedo values for UVA wavelengths determined in Simic et al. [31] at a low snow line of 800 m.

## 2. Materials and Methods

Haluza et al. [10] analyzed Austrian melanoma incidence data (1990–2010) by district and year, and found that melanoma incidence rates increase with altitude by as much as 30% per 100 m of the main capital of the respective district in which people are living, with about 50% higher rates in urban compared to rural districts. Investigations have shown that the increase in irradiance on a horizontal surface as a function of altitude is much smaller, and can account for only 2% of the effect [14]. This large discrepancy can therefore hardly be explained by the increase in irradiance alone, and requires an alternative explanation. It should be noted that irradiance on a horizontally-oriented surface is not a good indicator to determine the exposure of a human [20]. Instead, the multidirectional downwelling and upwelling UV radiation and a 3D-human model should be considered. Therefore, in the current study, we used the radiance (describing the radiant energy per unit solid angle and per unit area, thus taking into account the complex radiation field) to investigate if multidirectional UV exposure is partially responsible for increasing melanoma prevalence with altitude. 

Earlier investigations (e.g., by Diffey [32] and McKenzie et al. [33]) were based on the irradiance incident on surfaces, whereas the calculated exposure of a voxel model of a human takes into account the complex geometry of the radiation field as well as the geometry of a human body. We refer to a method to calculate biologically-weighted exposure by integrating the incident solar spectral radiance over all relevant parts of the human body [20].

**Figure 1:**
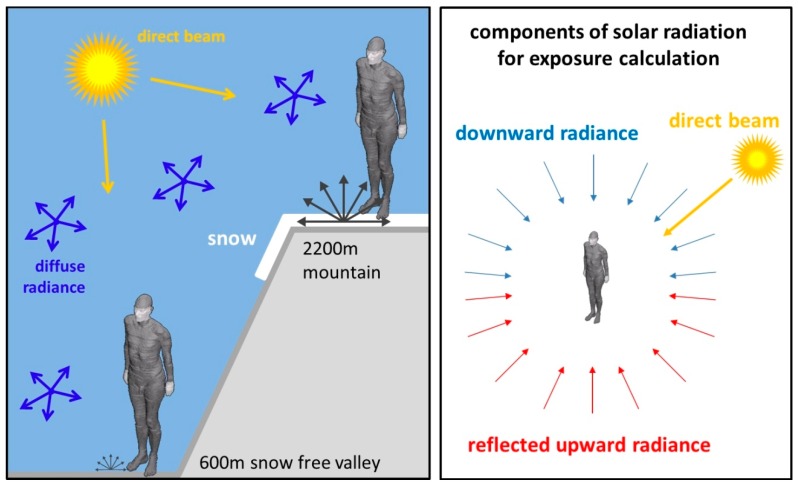
Illustration of the simulated locations. The exposure model takes into account the direct, diffuse, and reflected radiations, the complex geometry of a human body, and clothing. The albedo values of the location at 2200 m have been varied from 0.02 to 0.6. For a lower-altitude location at 600 m, an albedo of 0.02 was used.

In addition to the downwelling radiance and the direct beam of the sun already used in Seckmeyer et al. [20], we calculated the reflected upwelling radiance from the lower hemisphere for this investigation (see Figure 1). With the assumption that the snow-covered ground is a Lambertian surface, we can calculate the reflected upwelling radiance assuming a constant L (Lambertian surface). The radiance can be derived by the following Equation (1):

(1) $$\begin{array}{ccc} E_{upwelling}\left(\lambda \right) & = & \int_{Ω}L_{upwelling}\left(\lambda \right)cos\;\theta \;dΩ\;,\;with\;dΩ=sin\;\theta \;d\theta d\varphi \\ & = & \overset{2\pi }{\underset{\varphi =0}{\int }}\overset{\frac{\pi }{2}}{\underset{\theta =0}{\int }}L_{upwelling}\left(\lambda \right)cos\;\theta \;sin\;\theta \;d\theta \;d\varphi \\ & = & L_{upwelling}\left(\lambda \right)\;\times \;\;\pi \;\times \;sr \end{array}$$

where $E_{upwelling}\left(\lambda \right)$ is the spectral reflected irradiance, $L_{upwelling}\left(\lambda \right)$ is the spectral upwelling radiance, dΩ the solid angle, θ represents the zenith angle, and φ the azimuth angle. With the definition, $albedo=\frac{M_{G}}{E_{G}}$, where *E_G_* is the short-wave global irradiance and *M_G_* is the radiant exitance of the Earth’s surface in Wm^−2^ [34], $E_{upwelling}\left(\lambda \right)$ can be related to the global spectral downwelling irradiance ($Eglo_{downwelling}\left(\lambda \right)$) as in Equation (2):

(2) $$E_{upwelling}\left(\lambda \right)\;=Eglo_{downwelling}\left(\lambda \right)\;\times \;albedo$$

The spectral reflected upwelling radiance $L_{upwelling}\left(\lambda \right)$ is then given by Equation (3):

(3) $$L_{upwelling}\left(\lambda \right)\;=\;\frac{E_{upwelling}\left(\lambda \right)}{\pi \;\times \;sr}\;=\;\frac{Eglo_{downwelling}\left(\lambda \right)\;\times \;albedo}{\pi \;\times \;sr}$$

to calculate the human exposure, Seckmeyer et al. [20] combined the downwelling radiance with the geometry of the 3D-model seen from the upper hemisphere. Analogously, the reflected upwelling radiance is combined with the geometry of the 3D-model seen from the lower hemisphere (Figure 1). 

## 3. Results

### Hypothesis

In this investigation, the UV exposure of a human as defined in Seckmeyer et al. [20] is calculated for the wavelength range of 250–400 nm. Two hypothetical locations on 31 March 2016 with a latitude of 47° N are considered, one located in a snow-free valley at 600 m and one with varying albedo located on a mountain at 2200 m altitude (see Figure 1). Additionally, the irradiance is calculated for both locations. For the snow-free ground location, an albedo of 0.02 is used, which is typical for many surfaces in the UV wavelength region (e.g., grass) [35]. Since the albedo is influenced by a large surface area and there are objects with a lower reflectivity (e.g., rocks and trees) within that area, the assumption is made that the albedo extends to infinity, and that an effective albedo is used. For the mountain location, the effective albedo values of 0.02, 0.2, 0.4, and 0.6 were considered. The calculated spectra were weighted with the erythemal action spectrum. The exposure calculations presumed a human with winter clothing, where only hands and face are exposed (see Figure 1). 

**Figure 2:**
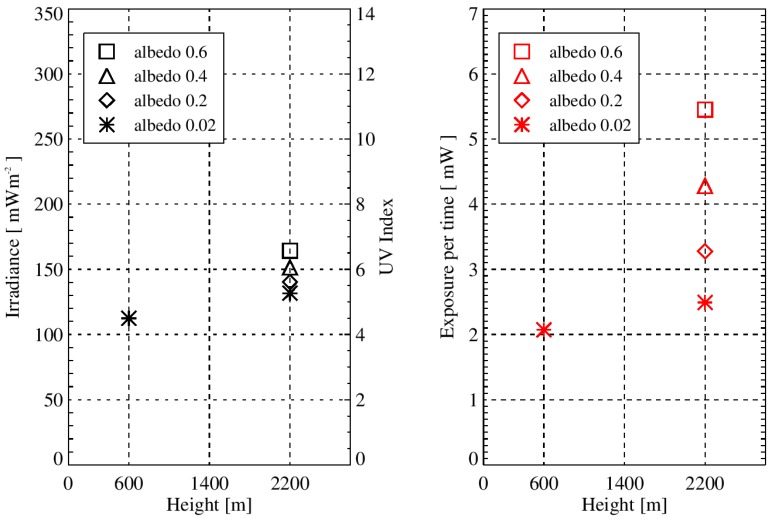
Simulated values of the irradiance (**left plot**) and the exposure of a human with winter clothing (**right plot**) for the valley and mountain locations are shown. The simulations were performed for a hypothetical location at 47° N for 31 March 2016 with a solar zenith angle (SZA) of 45°. The influence of the albedo is larger for the exposure (integrated radiance on a human) than for the irradiance.

Figure 2 depicts simulated values of the irradiance and the exposure for the valley location at 600 m, as well as for the mountain top location at 2200 m for 31 March 2016 with a solar zenith angle (SZA) of 45°. All mountain values are greater than the valley values. However, the albedo has a greater influence on the exposure than on the irradiance.

Gradients in % per 100 m have been calculated to compare the increase in exposure values with altitude to the increase in irradiance with altitude, and the melanoma incidence rates increase with altitude. The UV exposure and irradiance values are displayed in Figure 2, and the calculated gradients of the increase with altitude of these values are listed in Table 1. The gradients are calculated with Equation (4):

(4) $$gradient\;=\;\frac{\left(\frac{mountain\;value}{valley\;value}\right)\;-\;1}{\frac{\Delta \;altitude}{100\;m}}$$

It is evident that the gradients are increasing with higher albedo values on the mountain. Although the gradients of human exposure are always larger than those of irradiance, with a small albedo (e.g., 0.02), the ground reflection is minimal and the gradient of human exposure is therefore only about 1.2 times as large as the gradient in irradiance. However, for higher albedo values (e.g., 0.4 and 0.6), the gradient of the exposure is about 3 and 3.5 times as large, demonstrating that the ground reflection has an enormous impact on the total exposure. In addition, simulations have been performed for an urban valley location (with urban aerosol conditions instead of rural), where the total aerosol amount is higher than with rural settings. Consequently, there is an increased absorption of radiation by aerosols and thus lower exposure values at the valley location. The calculation of the gradient with an urban valley location and a rural mountain location resulted in a 15% per 100 m increase of human exposure in the case of a mountain top albedo of 0.6. This is about 50% higher than the gradient for a rural valley location (see Table 2). Larger gradients due to urban valley conditions equally occur for the irradiance. However, these gradients are still small.

**Figure 3:**
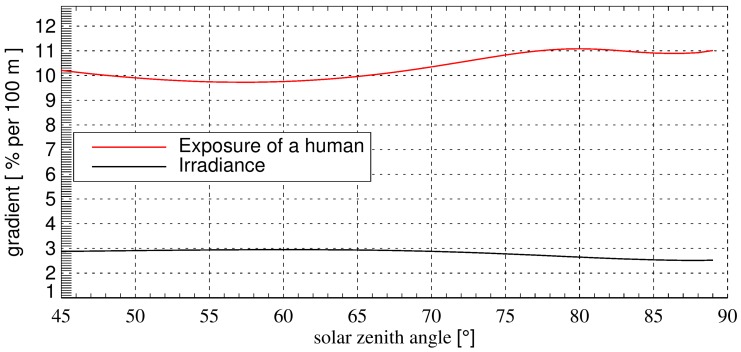
Gradients of valley (albedo = 0.02) to mountain (albedo = 0.6) values as increase with altitude in % per 100 m in dependence of the SZA. The gradients were calculated with Equation (4).

Since there are no routine measurements of surface albedo at Sonnblick observatory, Simic et al. [31] deployed an algorithm that uses routine observations of snow condition (snow height, time since last snowfall, and snow line) to estimate effective spectrally-invariant surface albedo in the UV range on a daily basis. Albedo values determined in this study ranged from 0.08 to 0.30 with snowline at 2500 m, and up to 0.55 to 0.75 with snowline at 1000 m. Based on these simulations and measurements, we use 0.6 as a constant albedo value for the mountain location at 2200 m altitude for an investigation of the solar zenith angle dependence on the radiation quantities (Figure 3). The calculations show that the diurnal variations of the gradients of the exposure and of the irradiance are small. The gradient of the exposure ranges between 9.7% and 11.1% per 100 m for SZA ranging between 45° and 89°.

## 4. Discussion

For this study, the simulations were calculated by the DISORT code of the UVSPEC model in the LibRadTran package [36]. Since two hypothetical locations were investigated, only basic parameters with conservative settings were used to create simple situations. These results show that under basic conditions, the gradients of the increase with altitude for human exposure are much greater than for literature values of the irradiance. In reality, the differences between the valley and mountain top atmospheric parameters could be larger, which would result in even greater gradients. The parameters with different values depending on location used in this study are listed in Table 3. The total ozone-column value for the mountain top is smaller than the valley value, due to the decrease of tropospheric ozone by 3.5 DU per 1000 m altitude [37]. For the investigation of a rural and urban valley location, the LibRadTran parameter Boundary layer aerosol was used [36]. 

As shown in this study, the irradiance on a horizontal surface should not be used to connect radiation changes with the increase in melanoma incidences with altitude. Instead, exposure may be used as it takes into account the complex geometry of a human body and radiation from multiple directions, including reflected radiation from the ground. 

We suggest further investigation using newly-developed techniques to measure the spectral radiance of more than 100 directions simultaneously [38]. With such a system, the downwelling and the ground-reflected upwelling radiation could be measured at different locations (e.g., valley and mountain locations) and used as input in the exposure model. Since such systems are not very mobile, additional data at numerous locations could be acquired using personal dosimeters. Personal dosimeters cannot replace spectroradiometer measurements, as the former often show large deviations from spectroradiometer measurements [39]. However, if dosimeters are carefully characterized and preselected, the use of dosimeters could extend the amount of data to estimate the exposure of a human in mountainous snow-free and snow-covered areas. In fact, dosimeter campaigns have already been performed, but rarely in mountainous regions. Siani et al. [40] conducted measurements with personal dosimeters in alpine sites by mounting dosimeters on the forehead of skiers. However, they measured with only one dosimeter and compared the measurements with irradiance measurements. Instead, to realistically estimate the human exposure, it would be necessary to use multiple dosimeters placed at different places on the human body to take the radiation from multiple directions into account simultaneously, including reflections from the ground.

## 5. Conclusions

In the current study, we demonstrated that upwelling radiation is a relevant factor that needs to be considered when investigating human exposure and its impact on skin cancer incidence. This is particularly important when the surface is covered with snow, thus causing a significant increase in the upwelling UV radiation. The increase in UV exposure with altitude shown here may help explain the increase in melanoma incidence with altitude, whereas the increase in irradiance with altitude leads to the incorrect conclusion that UV radiation plays a minor role for the altitude-related melanoma incidences. Additionally, human behavior is also a prominent factor that may determine the actual UV dose received (exposure integrated over time). A comparison of different valley locations showed lower exposure values with urban aerosol conditions due to higher total aerosol amounts. In contrast to two locations with rural aerosol settings, the calculated gradient for an urban valley location and a rural mountain location resulted in a 50% higher increase in exposure with altitude. This result, and high doses of intermittent UV exposure through recreational activities at snow-covered locations (e.g., skiing, swimming, sunbathing) may also help to explain higher incidence rates in urban districts compared to those in rural ones. Despite the complexity of assessing the real exposure of humans, differences in irradiance cannot explain the evidence in skin-cancer incidence with altitude. Instead, the exposure or radiance should be considered in further studies. 

## Figures and Tables

**Table 1:** Simulated values of the irradiance and the human exposure for the valley and mountain location are shown. Additionally, the gradients of the increase with altitude of the simulated values were calculated with Equation (4), where Δaltitude = 1600 m was used. The influence of the albedo is larger for the exposure of a human than for the irradiance.

Location	Irradiance		Exposure of a Human	
	Value $[\frac{mW}{m^{2}}]$	Gradient [%]	Value [mW]	Gradient [%]
Valley (Albedo 0.02)	112.46	-	2.07	-
Mountain (Albedo 0.02)	131.63	1.07	2.49	1.27
Mountain (Albedo 0.2)	140.25	1.54	3.28	3.64
Mountain (Albedo 0.4)	151.28	2.16	4.28	6.66
Mountain (Albedo 0.6)	164.19	2.88	5.45	10.21

**Table 2:** Comparison between gradients of rural and urban valley location values to mountain location values as increase in % per 100 m for human exposure. The gradients were calculated with Equation (4), where Δaltitude = 1600 m was used. Due to the urban aerosol conditions, the urban valley exposure value is lower compared to the rural valley value, resulting in larger gradients in the increase of the human exposure with altitude.

Location	Exposure of a Human (Rural Valley Location)		Exposure of a Human (Urban Valley Location)	
	Value [mW]	Gradient [%]	Value [mW]	Gradient [%]
Valley (Albedo 0.02)	2.07	-	1.59	-
Mountain (Albedo 0.02)	2.49	1.27	2.49	3.52
Mountain (Albedo 0.2)	3.28	3.64	3.28	6.60
Mountain (Albedo 0.4)	4.28	6.66	4.28	10.52
Mountain (Albedo 0.6)	5.45	10.21	5.45	15.12

**Table 3:** Parameter values used in this study.

Parameter	Valley Location	Mountain Top Location
Total Ozone Column	300	294.4
Horizontal Visibility	20 km	30 km
Boundary Layer Aerosol	Rural/Urban	Rural
